# CFTR and Anoctamin 1 (ANO1) contribute to cAMP amplified exocytosis and insulin secretion in human and murine pancreatic beta-cells

**DOI:** 10.1186/1741-7015-12-87

**Published:** 2014-05-28

**Authors:** Anna Edlund, Jonathan LS Esguerra, Anna Wendt, Malin Flodström-Tullberg, Lena Eliasson

**Affiliations:** 1 Unit of Islet Cell Exocytosis, Lund University Diabetes Centre, Department Clinical Sciences in Malmö, Lund University, Clinical Research Centre, SUS Malmö, Jan Waldenströms gata 35, SE 205 02 Malmö, Sweden; 2 Center for Infectious Medicine, Department of Medicine Huddinge, Karolinska Institutet, Stockholm, Sweden

**Keywords:** CFTR, Cystic Fibrosis, Diabetes, Insulin secretion, Islet of Langerhans, Beta-cell, Exocytosis

## Abstract

**Background:**

Mutations in the cystic fibrosis transmembrane conductance regulator (*CFTR*) gene lead to the disease cystic fibrosis (CF). Although patients with CF often have disturbances in glucose metabolism including impaired insulin release, no previous studies have tested the hypothesis that CFTR has a biological function in pancreatic beta-cells.

**Methods:**

Experiments were performed on islets and single beta-cells from human donors and NMRI-mice. Detection of CFTR was investigated using PCR and confocal microscopy. Effects on insulin secretion were measured with radioimmunoassay (RIA). The patch-clamp technique was used to measure ion channel currents and calcium-dependent exocytosis (as changes in membrane capacitance) on single cells with high temporal resolution. Analysis of ultrastructure was done on transmission electron microscopy (TEM) images.

**Results:**

We detected the presence of CFTR and measured a small CFTR conductance in both human and mouse beta-cells. The augmentation of insulin secretion at 16.7 mM glucose by activation of CFTR by cAMP (forskolin (FSK) or GLP-1) was significantly inhibited when CFTR antagonists (GlyH-101 and/or CFTRinh-172) were added. Likewise, capacitance measurements demonstrated reduced cAMP-dependent exocytosis upon CFTR-inhibition, concomitant with a decreased number of docked insulin granules. Finally, our studies demonstrate that CFTR act upstream of the chloride channel Anoctamin 1 (ANO1; TMEM16A) in the regulation of cAMP- and glucose-stimulated insulin secretion.

**Conclusion:**

Our work demonstrates a novel function for CFTR as a regulator of pancreatic beta-cell insulin secretion and exocytosis, and put forward a role for CFTR as regulator of ANO1 and downstream priming of insulin granules prior to fusion and release of insulin. The pronounced regulatory effect of CFTR on insulin secretion is consistent with impaired insulin secretion in patients with CF.

## Background

The cystic fibrosis transmembrane conductance regulator (CFTR) is a cAMP-regulated chloride channel that belongs to the family of ATP-binding cassette (ABC)-transporters [1]. As many other ABC-transporters, CFTR contains two membrane spanning domains (MSDs) and two nucleotide binding domains (NBDs) that interact with ATP. In addition, CFTR has a regulatory domain (R) that contains several phosphorylation sites. The chloride selective pore of the channel is formed by the MSDs, whereas the other domains control channel gating [1]. Apart from being an anion channel, CFTR has been suggested to act as a regulator of other proteins and ion channels [2] in similarity with other members of the family of ABC-transporters, such as the sulfonylurea receptor (SUR1).

CFTR is primarily present in epithelial cells in airways, intestine and in cells with exocrine functions. Mutations in the gene encoding the channel protein complex (*cftr*) cause the autosomal recessive disease cystic fibrosis (CF). In patients with CF the defective chloride transport through CFTR leads to production of thick viscous mucus caused by a disturbed ion and water transport across epithelial membranes. The aberrant secretory functions cause obstruction of the distal airways and intestine, pancreatitis and malabsorption [3].

With increasing lifespan many CF patients develop cystic fibrosis-related diabetes (CFRD). The mean age of CFRD onset is around 20 years and the prevalence varies between 30 to 50% depending on the population studied [4-7]. Despite an increased awareness of the clinical impact of CFRD surprisingly little is known about its etiopathogenesis. CFRD is associated with exocrine pancreatic insufficiency caused by duct obstruction and fibrosis, but not all patients with pancreatic insufficiency develop diabetes [8,9]. It has been suggested that the endocrine problem is, at least in part, mechanical [4,9]. Abnormalities in the islet architecture and beta-cell loss have been observed while alpha- and delta-cell mass have remained intact or even increased [10-13]. However, observations showing that CFRD often correlates with insulin deficiency and/or a significant lower first-phase insulin secretion (for example, in [8,9,14,15]), clearly indicate that other, beta-cell intrinsic factors, may play a causative role in CFRD. Moreover, many CF patients, also those without CFRD, have normal fasting plasma glucose levels, but suffer from postprandial hyperglycemia indicating that the beta-cells fail to respond upon increased insulin demand. A recent pilot study [16] performed on five CFRD patients with the G551D mutation having reduced/absent acute insulin response is in support of an insulin secretion defect in CFRD. One-month treatment with Ivacaftor, a newly developed CFTR potentiator, increased the insulin response to oral glucose in four out of the five patients. On a cellular level, data from insulin secreting HIT-cells support a role for an ATP-sensitive and cAMP-activated current in insulin secreting cells [17]. Boom *et al*. revealed that CFTR mRNA and protein are expressed by both alpha- and beta-cells in rats [18], but did not investigate the physiological importance of CFTR for the functions of these cells. In the present study, we have used murine and human pancreatic islets and single beta-cells to test the hypothesis that active CFTR is present in beta-cells and essential in regulating insulin secretion.

## Methods

### Ethical statement

Animal procedures were approved by the local ethics committee for use of laboratory animals in Malmö, Sweden. Human islet isolation from deceased donors and experimental protocols were approved by the ethics committee in Uppsala and Malmö, Sweden.

### Islet isolation and cell culture

Islets from 43 non-diabetic human donors (F/M 18/25, age 59 ± 1.3, body mass index (BMI) 25.5 ± 0.5 kg/m^2^, HbA1c 5.7 ± 0.04, days in culture 4.1 ± 0.3) were used for secretion measurements, patch-clamp experiments, qPCR and immunohistochemistry. For these experiments islets were hand-picked to ensure high purity. Human islets were provided by the Nordic Network for Clinical Islet Transplantation (Uppsala, Sweden) through the LUDC Human Tissue Laboratory. Female NMRI mice (Bolmholtgaard, Ry, Denmark) were sacrificed by cervical dislocation and islets were isolated by collagenase digestion and hand-picked prior to experiments. For patch-clamp measurements, human and mouse islets were dispersed into single cells as previously described [19].

### mRNA expression measured with RT-qPCR

Total RNA from human and mouse islets were prepared as described [20]. CFTR mRNA expression was measured by RT-qPCR using primers and probes from Taqman mRNA assays (Life Technologies, California, USA). Human CFTR: Hs00357011_m1 expression was normalized against human HPRT1: 4333768 F, while mouse CFTR: Mm00445197_m1 expression was normalized against mouse HPRT1: Mm00446968. RT-qPCR runs were performed for individual batches of human (*N* = 5) and mouse (*N* = 5) islets in triplicate wells of 384-well plate on a 7900 HT RT-PCR system (Applied Biosystems, California, USA).

### Immunocytochemistry

Human or mouse single cells were fixed and stained as described elsewhere [21]. Primary antibodies; Mouse monoclonal anti-CFTR (MATG-1061, RD-Biotech, France), Guinea pig monoclonal anti-insulin (Linco, Billerica, MA, USA), Guinea pig monoclonal anti-insulin (EuroDiagnostica, Malmö, Sweden) and rabbit polyclonal anti-Stx1A (Synaptic Systems, Goettingen, Germany). Secondary antibodies; guinea pig conjugated to Cy2, mouse conjugated to Cy3, guinea pig and rabbit conjugated to Cy5 (all from Jackson, UK). Immunofluorescence was detected with a confocal microscope (META 510, Zeiss, Germany) and unspecific binding of the secondary antibodies was excluded by parallel experiments in the absence of the primary antibodies. Localization of CFTR was analyzed as described elsewhere [22]. In short, the ratio between the mean fluorescent intensity in the plasma membrane region (P_1_) and the cytosolic region (P_2_) was measured using ZEN software (Zeiss, Jena, Germany). Pixel by pixel co-localization analysis was also performed using Zen software. The same laser setting was retained between experiments enabling comparison of experiments performed at different occasions.

### Patch-clamp recordings

EPC10 amplifier and 8.80 pulse software (HEKA, Lambrecht/Pfalz, Germany) was used to evoke and record whole-cell currents and changes in membrane capacitance on single mouse and human islet cells as described [19]. Current and capacitance measurements were performed at RT and 32 to 33°C, respectively. Extracellular solution contained: 118 mM NaCl, 20 mM TEACl, 5.6 mM KCl, 2.6 mM CaCl_2_, 1.2 mM MgCl_2_, 5 mM HEPES, 3 (current measurements)/5 (capacitance measurements) mM D-glucose pH 7.4 (NaOH), supplemented with 10 μM FSK, 200 μM 4,4'-Diisothiocyano-2,2'-stilbenedisulfonic acid (DIDS), 10 μM CFTR_inh_-172 (Sigma Aldrich, Sweden), 25 μM (current measurements) or 40 μM (capacitance measurements) GlyH-101 (Calbiochem, USA) [23,24] and 50 μM T16Ainh-AO1 (gift from A.S. Verkman, Department of Medicine, UCSF School of Medicine) as indicated. The intracellular solution contained: 125 mM CsOH, 125 mM Glutamate, 10 mM CsCl, 10 mM NaCl, 1 mM MgCl_2_, 3 mM Mg-ATP, 4 mM EGTA (current measurement)/0.05 mM EGTA (capacitance measurement), 5 mM HEPES and 0.1 mM cAMP (capacitance measurements) (pH 7.15 with CsOH).

### Insulin secretion measurements

Insulin secretion was measured in static batch incubations described previously [25]. Briefly islets were pre-incubated in 1 mM glucose for 30 minutes followed by 1 h incubation in extracellular solution with variable glucose concentration. Incubation was 15 min when 50 mM KCl was used as stimulator. The extracellular solution was supplemented with 10 μM FSK, 0.1 μM GLP-1 (Bachem, Weil am Rhein, Switzerland), 100 μM Tolbutamide (Sigma Aldrich, Stockholm, Sweden), 200 μM DIDS, 50 μM TMinh-AO1, 40 μM CFTRinh-172 and/or 50 μM GlyH-101 as indicated. Insulin secretion was measured using radioimmunoassay kit (mouse: Linco Research Inc.,; human: Millipore, Billerica, MA, USA).

### Transmission electron microscopy

Islets were collected after insulin secretion assay and prepared for electron microscopy, examined and analyzed as previously described [26]. The granule volume density (N_v_) and surface density (N_s_) were calculated using in-house software programmed in MatLab.

### Statistical analysis

Data are presented as mean ± SEM of N number of individuals or independent experiments and number of cells or biological replicates. Statistical significance was calculated using ANOVA or Student’s *t* test and *P* <0.05 was considered significant*.*

## Results

### CFTR-antagonists inhibit insulin secretion and an ATP-sensitive and cAMP-dependent current in mouse and human beta-cells

**Figure 1:**
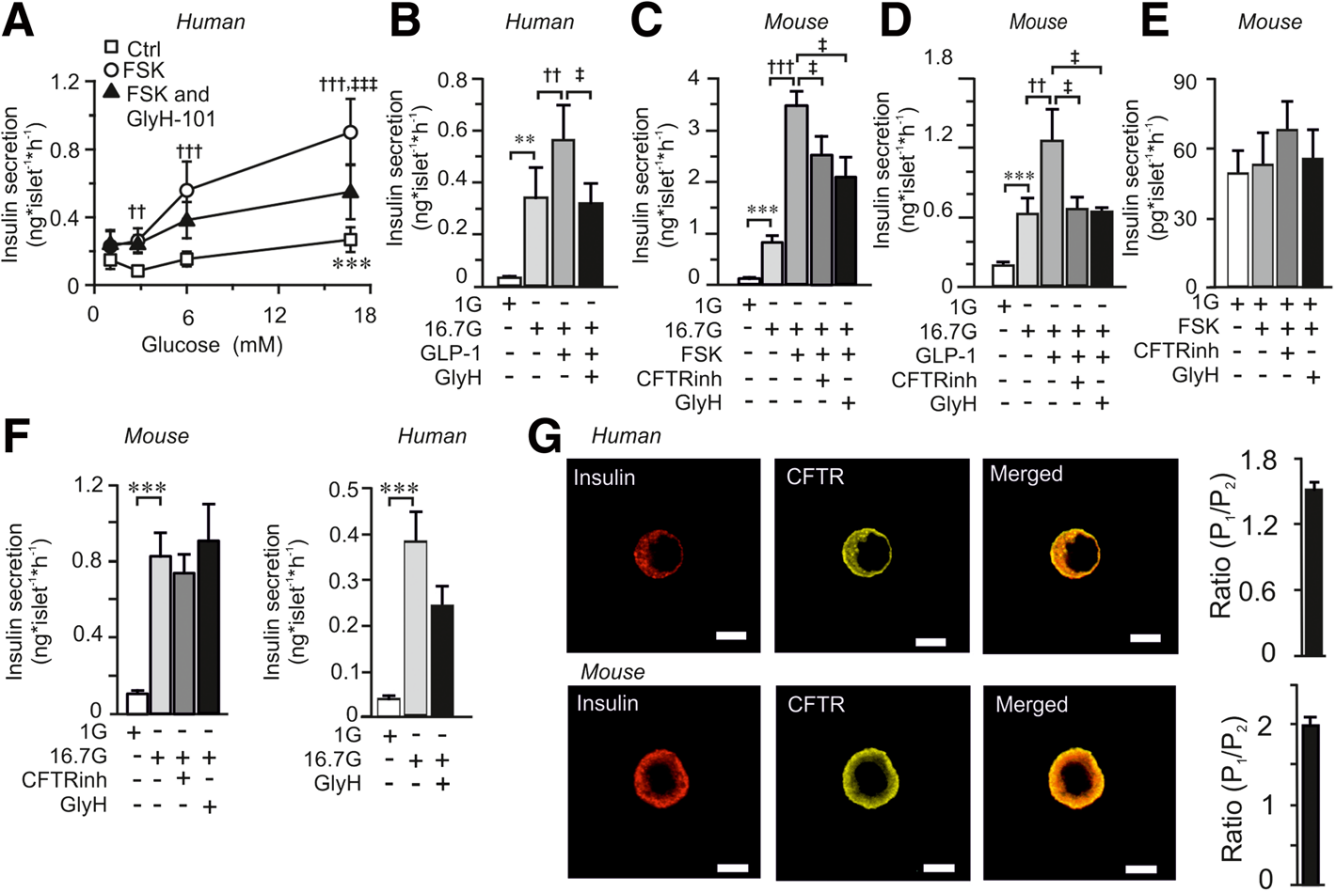
**Effect of CFTR inhibition on insulin secretion measured in isolated human and mouse pancreatic islets. (A)** Insulin secretion from human islets at different glucose in the absence or presence of forskolin (FSK) and GlyH-101 (GlyH) as indicated (n = 42 to 45, N = 11). **(B)** Insulin secretion from human islets at 1 mM glucose (1G) or 16.7 mM glucose (16.7G) in the absence and presence of GLP-1 and GlyH-101 as indicated (n = 17 to 19, N = 5). **(C-D)** Insulin secretion from mouse islets at 1 mM glucose (1G) or 16.7 mM glucose (16.7G) in the absence or presence of FSK, GLP-1, CFTRinh-172 (CFTRinh) and GlyH-101 (GlyH) as indicated (n = 12 to 21, N = 4 to 9). **(E)** Insulin secretion from mouse islets at 1 mM glucose (1G) in the absence or presence of FSK and inhibitors as indicated (n = 9–12, N = 4). **(F)** Insulin secretion from mouse (left) and human (right) islets in the absence of FSK to demonstrate the lack of effect of the inhibitors (mouse: n = 10, N = 5; human n = 12, N = 3). **(G)** Localization of CFTR (yellow) and insulin (red) in fixed single islet cells (left) from human (top) and mouse (bottom)**,** detected using confocal immunocytochemistry. Scale bar 5 μm. Images are representative of 37 beta-cells from three human donors and 23 beta-cells from three mice. Ratio of the fraction of CFTR (right) in the plasma membrane region (P_1_) as compared to the cytosolic region (P_2_) for human (top) and mouse (bottom) beta-cells. Data are presented as mean ± SEM. ^***^P <0.001 16.7 G *vs* 1 G, ^††^*P* <0.01 FSK or GLP-1 *vs* respective G alone, ^†††^*P* <0.001 FSK *vs* respective G alone and ^‡^*P* <0.05 CFTRinh or GlyH *vs* 16.7 G and FSK alone, ^‡‡‡^*P* <0.001 GlyH *vs* 16.7G and FSK alone.

To investigate if CFTR affects beta-cell function, the influence of CFTR on insulin secretion was measured from isolated human and mouse pancreatic islets subjected to glucose-induced insulin secretion assays. In human islets, glucose caused a dose-dependent increase in insulin secretion that was further enhanced by the cAMP-increasing agent FSK. The stimulatory action of FSK at 16.7 mM glucose was significantly reduced in the presence of the CFTR-antagonist GlyH-101 (Figure 1A). Next we investigated the effect of a more physiological elevator of cAMP, the incretin hormone GLP-1. Like FSK, GLP-1 enhanced insulin secretion at 16.7 mM glucose in human islets, an effect that was completely inhibited by GlyH-101 (Figure 1B). In mouse islets, both FSK and GLP-1 amplified insulin secretion stimulated by glucose was reduced in the presence of GlyH-101. Moreover, CFTRinh-172, another antagonist against CFTR, also decreased both FSK and GLP-1 enhanced insulin secretion (Figures 1C, D). Both inhibitors are specific for CFTR, and whereas CFTRinh-172 binds to the intracellular domain of CFTR [23] and thereby closes the channel, GlyH-101 is an open-channel blocker [24]. It was confirmed that the antagonists had no significant effect on insulin secretion at 1 mM glucose (Figure 1E) or in presence of 16.7 mM glucose in the absence of FSK or GLP-1 (Figure 1F). There was, though, a tendency towards a reduction in insulin secretion by GlyH-101 in the presence of 16.7 mM glucose alone in the human islets. This might be due to a higher increase in cAMP caused by glucose. It has been demonstrated that glucose increases cAMP levels in both mouse [27] and human [28] beta-cells. However, why the increase in cAMP generated by glucose might be higher in human islets is not clear but could be explained by the higher number of alpha-cells in the human islets [29,30]. cAMP is a potent second messenger that promotes insulin secretion by several mechanisms [19,31]. Here we establish that CFTR has an essential and complementary function in cAMP-amplified insulin secretion. In parallel to these findings, we found expression of *CFTR* mRNA (data not shown) and CFTR protein in both human and mouse islets (Figure 1G).

**Figure 2:**
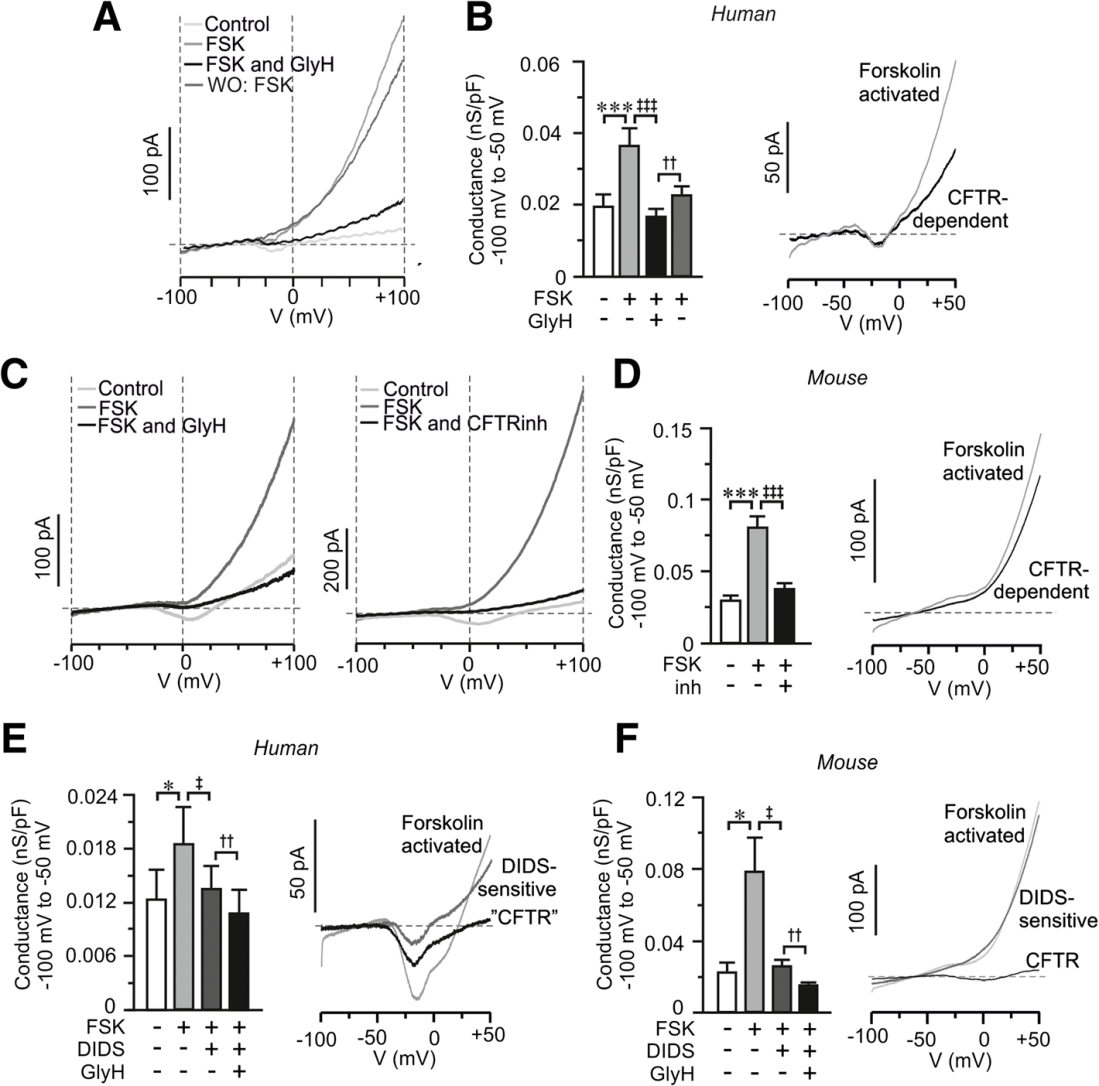
**cAMP-activated chloride currents in human and mouse beta-cells. (A)** Currents measured in a single human beta-cell after stimulation with voltage ramps in the absence (Ctrl, light gray) and presence of forskolin (FSK; gray), in the simultaneous presence of FSK and GlyH-101 (FSK and GlyH; black) and after wash-out of GlyH-101 to recover the FSK-activated current (WO: FSK; dark gray). Current ramps were applied before and every fourth minute after the application of FSK until a steady state was achieved. **(B)** Bar graph of the membrane conductance at negative voltages (left; n = 7 to 17, N = 3) and graph of calculated FSK-activated and CFTR-dependent current (right; Mean of n = 7 cells) from data in A. **(C)** Same as in A, but experiments where performed on mouse beta-cell. GlyH-101 (GlyH: black trace) and CFTRinh-172 (CFTRinh, black) was added to the left and right, as indicated. **(D)** As in B, but membrane conductance (left) was calculated from data in C (n = 10 to 17, N = 8). The mean result was combined for both CFTR-inhibitors (Inh). The calculated FSK-activated and CFTR-dependent current to the right is a mean from 10 cells. **(E)** As in A, but the effect of 4,4'-Diisothiocyano-2,2'-stilbenedisulfonic acid (DIDS) was investigated (n = 6, N = 2). Calculated FSK-activated, DIDS-sensitive and CFTR-currents shown to the right are mean of n = 5 cells. **(F)** Same as in E, but the membrane conductance (left) was calculated from measurements in mouse beta-cells (n = 9, N = 6) and the calculated current to the right is the mean from n = 8 cells. Data are presented as mean ± SEM. ^*^P <0.05, ^***^P <0.005, ^‡^*P* <0.05, ^‡‡‡^*P* <0.005, ^†^*P* <0.01 and ^††^*P* <0.01.

The presence of active CFTR channels in pancreatic beta-cells was investigated on single cells using the patch-clamp technique in the standard whole-cell configuration. The pipette solution contained sodium and calcium ions in order to determine the cell-type by sodium channel inactivation properties [32]. A voltage-ramp protocol from −100 mV to +100 mV was applied before and every fourth minute after the addition of FSK (10 μM) until steady state was achieved (Figure 2). In the absence of FSK the current flow was minimal, whereas the increase in intracellular cAMP induced by FSK activated a non-linear outward rectifying current. In human and mouse beta-cells, the cAMP-activated current was significantly inhibited by the CFTR-inhibitors (Figure 2A-D). The current inhibited by CFTR-inhibitors (CFTR-dependent) constitute 47 ± 15% (n = 7) and 57 ± 7% (n = 10) of the FSK-activated current at negative potentials, in human and mouse beta-cells, respectively.

In addition to the ion channel function, CFTR has been attributed a role as regulator of other ion channels and proteins, such as other chloride channels [2,33]. To investigate the possibility that CFTR regulates the function of other chloride channels we used the non-specific chloride channel blocker DIDS that blocks a wide variety of chloride channels, while CFTR is insensitive to this antagonist [34,35]. The cAMP-stimulated current, in human and mouse beta-cells, was significantly reduced by DIDS (Figure 2E, F). The presence of active CFTR channels was proven by a significant reduction in current conductance in the simultaneous presence of GlyH-101 and DIDS as compared with DIDS alone (Figure 2E, F). The DIDS-sensitive component constituted 38 ± 10% and CFTR 37 ± 15% (n = 5) of the FSK-activated current at negative potentials (−100 to −50 mV) in human beta-cells. In the mouse, the DIDS-sensitive current was 41 ± 12% and CFTR 29 ± 9% (n = 8) of the FSK-activated current. From these measurements the CFTR conductance was estimated to be 2.8 ± 0.6 pS/pF in human (n = 5; N = 3) and 12 ± 4.0 pS/pF in mouse (n = 8; N = 5) beta-cells at negative potentials (−100 mV to −50 mV). Taken together these data indicate that 1) the cAMP-induced chloride current has one DIDS-sensitive component and one part sensitive to CFTR inhibition, 2) the current through CFTR in beta-cells is small, and 3) CFTR likely acts as a regulator of DIDS-sensitive chloride channel(s).

### CFTR likely regulates the chloride channel Anoctamin 1 (ANO1)

**Figure 3:**
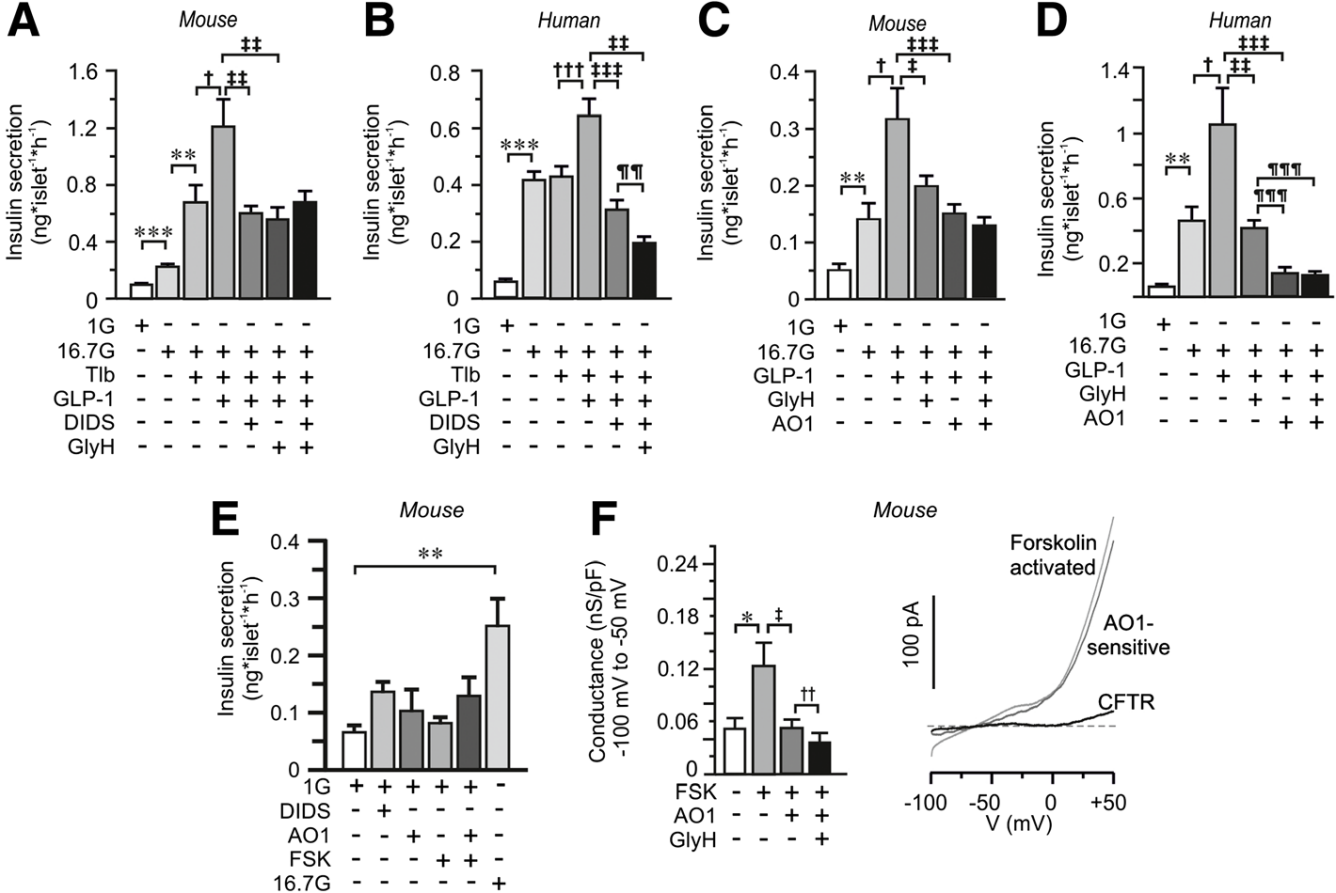
**The combined effect of chloride channel blockers on GLP-1 enhanced GSIS. (A)** Insulin secretion from mouse islets after 1 h incubation in 1 mM glucose (1G), 16.7 mM glucose (16.7G), tolbutamide (Tlb), GLP-1, DIDS- and GlyH-101 as indicated (n = 12 to 20, N = 4). **(B)** The same as in A, but insulin secretion was measured from human islets (n = 20 to 28, N = 5). **(C)** As in A, but the impact of the anoctamin 1 inhibitor TM16Ainh-AO1 (AO1) on GLP-1 enhanced insulin secretion was investigated in mouse islets (n = 14, N = 3). **(D)** As in C, but experiments were performed on human islets (n = 4, N = 1). **(E)** As in C, but the effects of forskolin (FSK) and AO1 was investigated at 1 mM glucose as indicated. Data are presented as mean ± SEM., ^**^P <0.01, ^***^P <0.005, ^†^*P* <0.05, ^†††^*P* <0.005, ^‡^*P* <0.05, ^‡‡^*P* <0.01, ^‡‡‡^*P* <0.005 ^¶¶^*P* <0.01. **(F)** Bar graph of membrane conductance at negative (left) membrane potentials in the absence and presence of FSK, TM16Ainh-AO1 (AO1) and GlyH-101 (GlyH) (n = 6, N = 4). Calculated FSK-activated, AO1-sensitive and CFTR-currents shown to the right are mean of n = 5 cells. Data are presented as mean ± SEM. ^*^P <0.05, ^‡^*P* <0.05, ^‡‡^*P* <0.01.

The suggestion that CFTR can act as a regulator upstream of other chloride channels involved in the regulation of insulin release was verified through the investigation of the combined effect of DIDS and GlyH-101 on insulin secretion. The experiment was conducted in the presence of tolbutamide to circumvent that DIDS might affect the ATP-dependent potassium channel [36]. At high concentrations tolbutamide can inhibit CFTR currents [37], but at the concentration used here (100 μM) the major influence is on the closure of the ATP-dependent potassium channel. This was confirmed by insulin secretion measurements showing that tolbutamide increased (rather than decreased) insulin secretion at 16.7 mM glucose in mouse islets, while no further stimulation of insulin secretion was observed in human islets (Figure 3A, B). This is most likely due to the different sensitivity of glucose in mouse and human islets and in line with previous results [38,39]. In the presence of tolbutamide, GLP-1-enhanced glucose-stimulated insulin secretion was significantly reduced in the presence of DIDS (Figure 3A, B), in both mouse and human islets. The addition of GlyH-101 in the continued presence of DIDS did not further reduce the secretory response in mouse beta-cells as compared to DIDS alone (Figure 3A), suggesting that CFTR regulates a DIDS-sensitive chloride channel.

Recently, it was suggested that channels from the family of Anoctamins (ANO1-10) are controlled by CFTR [40,41]. The first member in this family, ANO1, is a voltage- and calcium-dependent chloride channel localized in the plasma membrane that has been suggested to interact with CFTR to control chloride conductance in epithelial cells [41]. Interestingly, *ANO1* gene expression has been measured in rat and human islets [42,43]. To investigate the possible combined involvement of ANO1 and CFTR in the regulation of insulin secretion we performed insulin secretion measurements in mouse and human islets in the presence or absence of specific blockers of the respective channel. Indeed, insulin secretion enhanced by GLP-1 was reduced by TM16Ainh-AO1 (AO1), a specific blocker of ANO1 [44], and in the combined presence of GlyH-101 and AO1 the reduction in GLP-1 enhanced glucose-stimulated insulin secretion was not significantly different from the reduction observed after application of either of them alone (Figure 3C, D). In human islets, the inhibitory effect of AO1 was significantly more potent than GlyH-101 alone, but, as in the mouse, inclusion of GlyH-101 in the simultaneous presence of AO1 did not cause an additive decrease in GLP-1 amplified insulin secretion at 16.7 mM glucose (Figure 3D). It was confirmed that AO1 did not have any inhibitory effect at 1 mM glucose (Figure 3E).

The presence of ANO1 chloride current in beta-cells was measured by patch-clamp recordings on single mouse beta-cells using the standard whole-cell mode and the ramp-protocol as used above. It was obvious that the FSK-activated current was reduced by AO1, and the conductance was decreased (Figure 3F). A small, but significant, further reduction in conductance was obtained in the simultaneous presence of AO1 and the CFTR-inhibitor GlyH-101 (Figure 3F). The AO1-sensitive current was calculated to represent 50 ± 10% and CFTR 30 ± 5% of the FSK-activated current at negative potentials (n = 5). The CFTR conductance was estimated to 14 ± 3 pS/pA (n = 5). Interestingly, these are similar to values obtained from the DIDS-experiments in Figure 2 (12 pS/PA and 29%). This experiment demonstrates that the blockers AO1 and GlyH-101 most likely act on separate channels (Figure 3E). Hence, the lack of an additive effect on secretion (Figure 3C, D) would suggest that CFTR interacts with ANO1 in regulating insulin secretion.

### Inhibition of CFTR results in decreased exocytosis in mouse and human beta-cells

**Figure 4:**
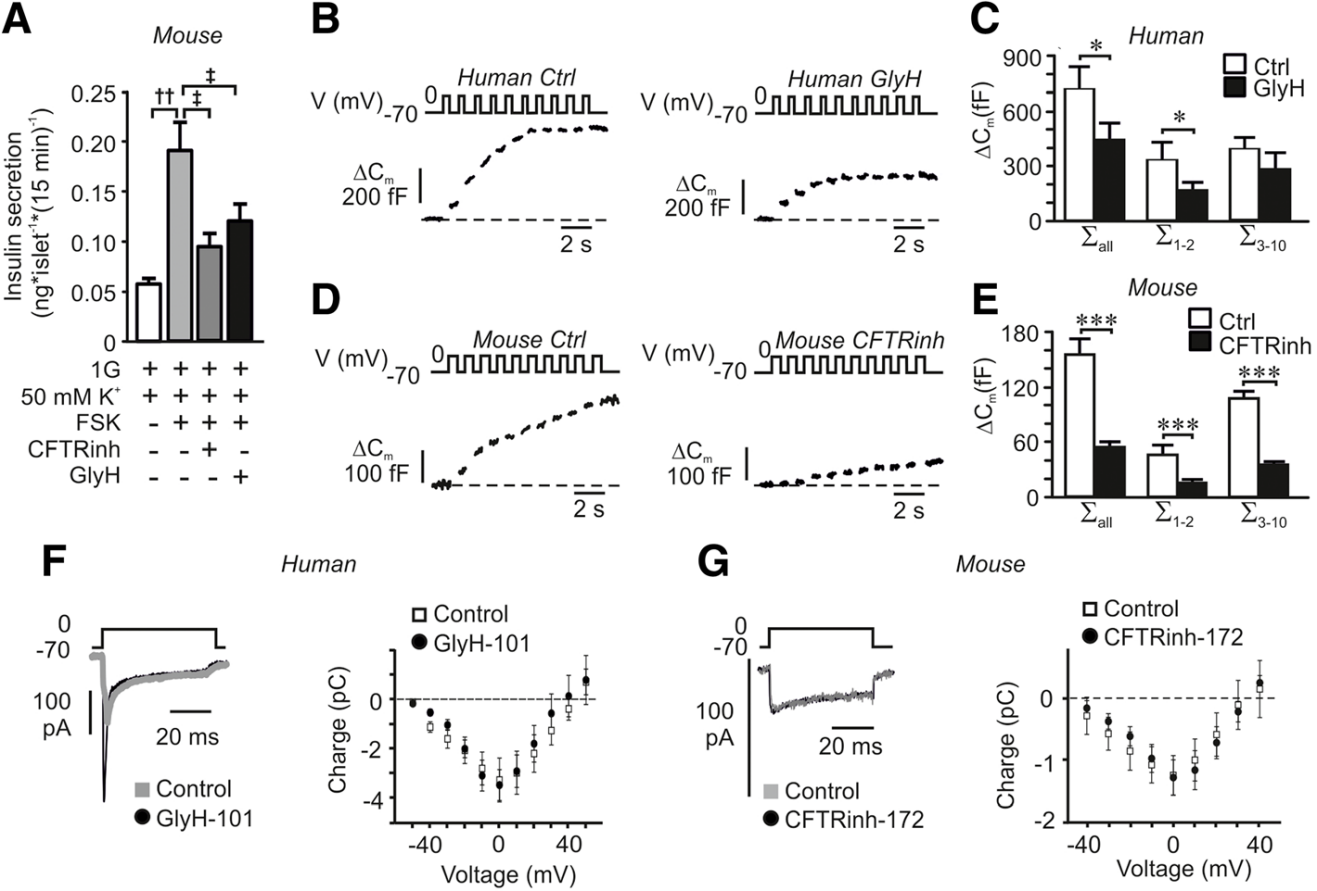
**Effect of CFTR inhibition on depolarization evoked secretion and exocytosis. (A)** Insulin secretion from mouse islets measured after 15 minutes incubation at 1 mM glucose (1G) in the presence of 50 mM KCl (K), 10 μM forskolin (FSK), CFTR-inh172 (CFTRinh) and GlyH-101 (GlyH) as indicated. Data are presented as mean ± SEM of n = 15 to 17, N = 4. ^††^*P* <0.01, ^‡^*P* <0.05. **(B)** Exocytosis in single human beta-cells measured as an increase in membrane capacitance (ΔCm; bottom left) under control conditions (Ctrl; left) and after 10 minutes pre-incubation with GlyH-101 (GlyH; right). Experiments were conducted in the presence of cAMP in the intracellular solution. **(C)** a summary of data in B (Ctrl; n = 6 and GlyH; n = 4). *P <0.05. Data are presented as the increases in membrane capacitance evoked by all 10 pulses of the train (∑_all_), the two first pulses (∑_1–2_) or the latter eight pulses (∑_3–10_). **(D)** As in B, but the increase in membrane capacitance was measured on single mouse beta-cells and CFTRinh-172 (CFTRinh) was used as an inhibitor of CFTR. **(E)** Summary of data in D (Ctrl; n = 10 and CFTRinh-172; n = 7). ***P <0.001. Data are presented as in C. **(F)** Inward voltage-dependent current in a single human beta-cell in the presence and absence of GlyH-101 (left) and charge-voltage relationship of voltage-dependent currents (right). Data are mean ± SEM of 10 control experiments and 5 experiments in the presence of GlyH-101. **(G)** As in F, but experiments were conducted on mouse beta-cells (Control; n = 7 and CFTRinh-172; n = 11).

We were next interested in investigating mechanisms by which CFTR and ANO1 influence insulin secretion. Insulin secretion comprises a cascade of events often referred to as the “stimulus-secretion-coupling” [45]. Briefly, glucose uptake and metabolism yields ATP, leading to depolarization of the plasma membrane, opening of voltage sensitive calcium channels and increased levels of intracellular calcium resulting in exocytosis of insulin granules. Theoretically, CFTR could be involved in any of these steps. Indeed, it has been suggested that an ATP-sensitive and cAMP-activated chloride current influences the membrane depolarization and electrical activity of insulin secreting cells [17]. We found that CFTR-inhibition reduced insulin secretion under conditions independent of cellular metabolism and membrane depolarization (Figure 4A), suggesting a possible function of CFTR in the downstream exocytotic process. To elaborate further on this, capacitance recordings were performed on single beta-cells in the presence of ATP and cAMP in the intracellular solution. Exocytosis, evoked by a train of 10 500-ms depolarizations from −70 mV to 0 mV, was significantly reduced in human beta-cells pre-incubated with GlyH-101 (Figure 4B, C). The reduction in membrane capacitance increase was most prominent during the first two depolarizations reflecting rapid exocytosis of primed granules [46]. The exocytotic response in mouse beta-cells was likewise reduced after CFTR-inhibition (Figure 4D, E). Next, effects on voltage-dependent calcium currents were investigated. The current was evoked by 50-ms depolarizations from −70 mV to voltages between -50 and +50 mV in single human or mouse beta-cells and in the presence and absence of GlyH-101 or CFTRinh-172, respectively (Figure 4F, G). The intracellular solution was supplemented with cAMP to activate CFTR. The currents comprise a rapid sodium current followed by a more slowly opening calcium current. The charge was measured as the integral of the current and reflected the influx through the calcium channels. From the data we could conclude that the calcium current was not affected by CFTR inhibition (Figure 4F, G), suggesting a direct effect on the exocytotic machinery [21]. Interestingly, DIDS has earlier been demonstrated to reduce insulin exocytosis, an effect that has been coupled to intragranular ClC3 chloride channels and priming of the insulin granules [47], implying that CFTR can act by controlling ANO1 to influence priming and exocytosis. Depolarization-evoked exocytosis of primed granules is hypothesized to correspond to first phase insulin secretion [46], thus suggesting an important role of CFTR at this stage. Indeed, patients with CF exhibit reduced first phase insulin secretion [9,15,16].

**Figure 5:**
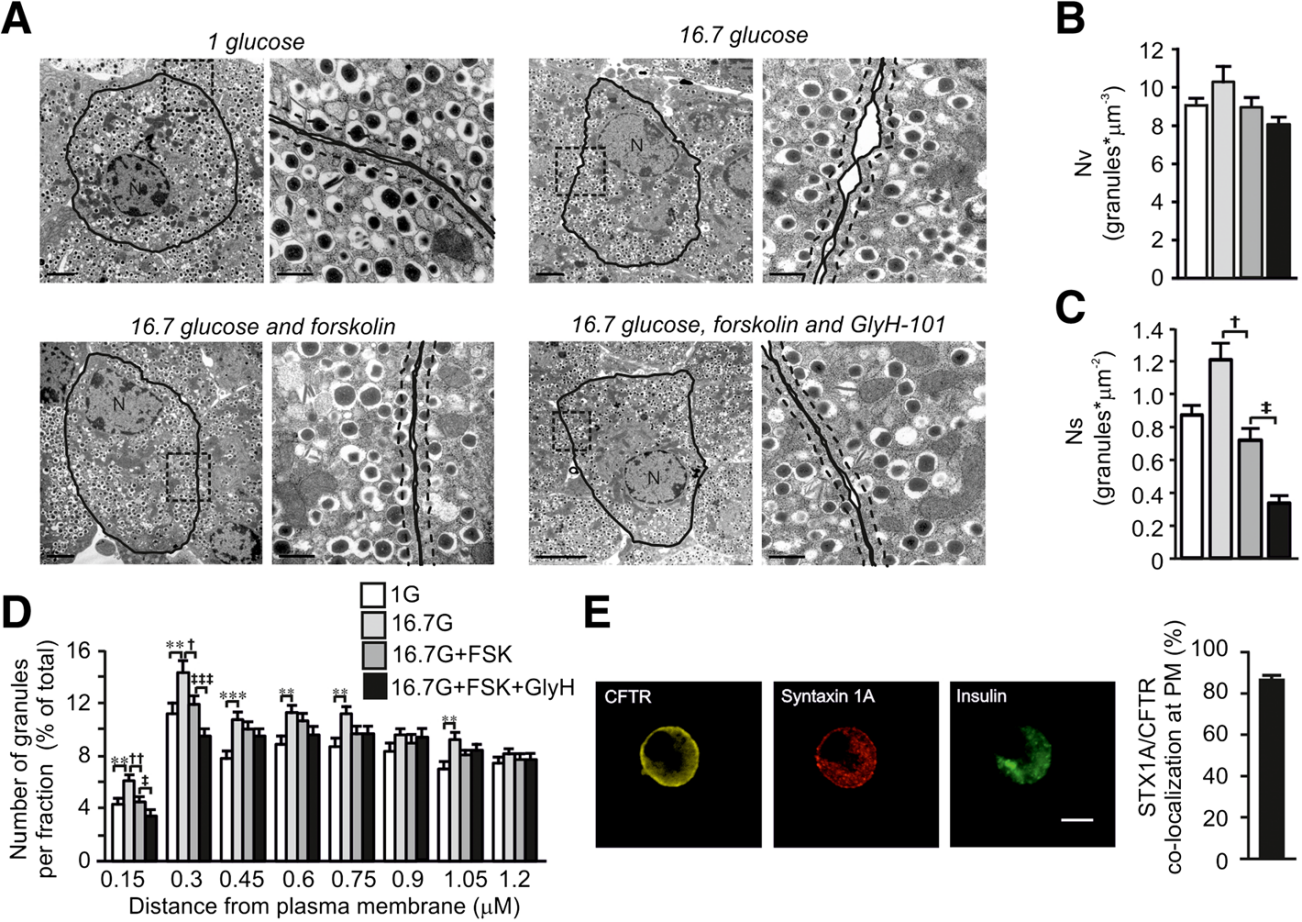
**Consequences on ultrastructural parameters after CFTR inhibition. (A)** Electron micrographs of a single beta-cell within an islet after incubation for one hour as indicated. The area within the dotted rectangle in the left image is highlighted to the right. The plasma membrane is indicated by a black solid line. The granules where defined as docked when the center of the granule was located within 150 nm from the plasma membrane (dashed line). N = nucleus. Scale bars: 2 μM (left) and 0.5 μM (right). **(B)** Bar graph of the total number of granules measured as volume density (N_v_; granules*μm-3) after incubation in 1 mM glucose (1 G), 16.7 mM glucose (16.7 G), 10 μM forskolin (FSK) and 50 μM GlyH-101 (GlyH) as indicated in the color coding in D. **(C)** Bar graph of the docked granules as estimated by the surface density (N_s_; granules*μm^−2^) under the different conditions as in D. **(D)** Relative distribution of granules at distance fractions from the PM. The distance at the x-axis is the upper border of each fraction. Color-coding for the different conditions is at the top right (n = 43 to 45 cells, N = 3 animals per condition). For B-D, data are presented as mean ± SEM. ^**^P <0.01 16.7 G *vs* 1 G, ^***^P <0.001 16.7 G *vs* 1 G ^†^*P* <0.05 FSK *vs* 16.7 G alone, ^††^*P* <0.01 FSK *vs* 16.7 G alone, ^‡^*P* <0.05 GlyH *vs* 16.7 G and FSK alone, ^‡‡‡^*P* <0.001 GlyH *vs* 16.7 G and FSK alone. **(E)** Localization of CFTR (yellow), syntaxin 1A (red) and insulin (green) in fixed single human islet cells detected using confocal immunocytochemistry (left). A bar graph describing the measured co-localization in the plasma membrane is shown to the right. The images are from a representative cell out of 39 from two human donors.

The above results prompted us to investigate granular docking using TEM, since docking of the granules to the plasma membrane is vital for exocytosis. Mouse islets were subject to incubation in 1 mM glucose, 16.7 mM glucose in the absence and/or presence of FSK and GlyH-101 prior to fixation (Figure 5A). We performed ultrastructural analysis and estimated the granule volume density per cell, N_v_, and the surface density per cell, Ns, from the micrographs. N_v_ and N_s_ are proportional to the total number of granules and the number of docked granules, respectively. The total number of granules within the beta-cells was not changed between islets treated under the different conditions (Figure 5B). Glucose significantly increased the docked pool (N_s_) in agreement with previous observations that glucose enhances granule refilling and mobilization [48]. Addition of FSK caused a reduction in the number of docked granules, confirming earlier electrophysiological observations that mobilization is rate-limiting [49]. Finally, the docked pool of granules was significantly reduced in beta-cells incubated with GlyH-101 and FSK, as compared to FSK alone (Figure 5C). Moreover, it is evident from the analysis that only the fraction of granules within 300 nm from the plasma membrane is affected by inhibition of CFTR (Figure 5D). This is in accordance with a role for CFTR in docking and priming of insulin granules.

CFTR has been shown to act through additional mechanisms, one being interaction with the SNARE-protein syntaxin 1A [50,51], a protein crucial for beta-cell granule docking and exocytosis [46]. To investigate the possibility that CFTR interacts with this SNARE-protein, we investigated the distribution of CFTR and syntaxin 1A, and found that CFTR was co-localized with syntaxin 1A in the plasma membrane of human beta-cells (86 ± 2% co-localization, n = 39; N = 2; Figure 5E).

## Discussion

Chloride channels play key roles in pancreatic beta-cells [17,52-54], but the exact identity of all chloride channels involved remains to be established. A previous study indicated that endocrine cells in the rat pancreas express the mRNA encoding CFTR [18]. Here we provide novel data adding CFTR to the list of chloride channels having important functions in human and mouse beta-cells [17,52-54], and suggest that CFTR acts upstream of ANO1 [2,33,41] to control insulin secretion.

In a previous study, Kinard and Satin [17] measured an ATP- and cAMP-dependent chloride current in insulin secreting cells that could be activated under hypotonic conditions. The current was termed I_Cl,islet_ and had similar properties in terms of size and reversal potential as the chloride current obtained here in the presence of glucose and FSK/GLP-1 (Figure 2). It was suggested [17] that the I_Cl,islet_ contributes to cAMP-dependent depolarization at negative membrane potentials although the exact identity of the channel was not described. It has been suggested that the channel is a volume-regulated anion channel (VRAC; [55]), due to similarities with I_Cl,islet_ in terms of activation and electrophysiological properties. Our focus was CFTR and we did not investigate the presence of a current sensitive to cell swelling. However, our data do not rule out the presence of VRAC. Whereas VRAC is suggested to enhance electrical activity, we hypothesize that the main function of CFTR/ANO1 is in the control of exocytosis. We confirm that activation of the ATP- and cAMP-dependent chloride current contributes to a small depolarization at potentials below the equilibrium potential for chloride, when the flux of chloride ions is outward from the cell comparable to the depolarization obtained by cAMP on electrical activity measured on whole islets [56,57].

The measured current conductance in the presence of FSK varies among different batches of cells investigated and amounts between approximately 80 and 120 pS/pF and approximately 20 and 40 pS/pF in mouse beta-cells (Figures 2B, F and 3F) and human beta-cells (Figure 2B, E), respectively. Biological variation is common in studies on primary tissue and human primary tissue in particular. However, more importantly, the estimated CFTR conductance after blockade of other chloride-currents using DIDS or by blocking ANO1 becomes the same (13 pS/pF and 12 pS/pF, respectively), strongly supporting the presence of a CFTR conductance. Although small, the impact on insulin secretion is large, suggesting that CFTR has a function upstream of many other cAMP-activated processes involved in insulin secretion [19,27,49,56,58].

We provide evidence suggesting that the main consequence of the cAMP-activated chloride current is unrelated to membrane depolarization (Figure 4A). More specifically our studies point to a role of CFTR and ANO1 in cAMP augmented calcium-dependent exocytosis. Indeed, both FSK- and GLP-1-enhanced, glucose-stimulated insulin secretion is reduced by CFTR-inhibitors. We observed that the inhibitors are more potent on GLP-1 than on FSK-stimulated secretion. This we mainly attribute to the fact that FSK increases cAMP to a higher level than GLP-1 in islets (see, for example, [19]). The participation of CFTR in the exocytotic process is supported by the capacitance measurements showing that exocytosis is blocked by CFTR-inhibitors in both human and mouse beta-cells (Figure 4). Exocytosis is a calcium-dependent process and the reduced exocytotic response observed in the presence of the CFTR antagonists might be explained by a reduced calcium current [59], but this was proven not to be the case (Figure 4F, G). Based upon our observations from insulin secretion and electrophysiological ramp-protocol measurements we instead hypothesize that CFTR through ANO1 act directly on exocytosis. Indeed, we found CFTR to co-localize with the SNARE-protein syntaxin 1A (Figure 5E) as demonstrated in other tissues [50,51]. This suggests that CFTR, as syntaxin 1A, is part of the exocytotic machinery.

From our novel results we suggest that CFTR plays a key role in priming of the insulin granules; this is evident from the capacitance measurements and the electron micrographic data. The increase in membrane capacitance evoked by a train of membrane depolarizations initiates exocytosis of release-ready (primed) granules by the first two depolarizations, whereas the latter depolarizations enable granules within a larger reserve pool to be released [46]. Our data demonstrate a pronounced reduction in exocytosis evoked during the two first depolarizations (Figure 4B-E). Hence, CFTR can be suggested to have its main function in the priming of insulin granules. Moreover, the ultrastructural analysis revealed that the number of granules in close vicinity to the plasma membrane (<300 nm) was reduced after CFTR inhibition (Figure 5D). The exact molecular mechanism by which CFTR contributes to granular priming is hitherto unknown. Interestingly, DIDS has earlier been demonstrated to reduce insulin exocytosis, an effect that has been coupled with intragranular ClC3 chloride channels and priming of the insulin granules [47]. A role for ClC3 in insulin granular priming and exocytosis has also been proven by knock-out animals [54]. Here we demonstrate that CFTR inhibition reduces exocytosis to the same extent as has earlier been demonstrated for DIDS [47] and removal of ClC3 [54]. As DIDS is a chloride channel blocker inhibiting the current through most chloride channels except for CFTR, the ANO1 current is likely reduced by this treatment (compare Figures 2F and 3F). Our insulin secretion measurements and chloride current measurements suggest that CFTR acts on ANO1. The mechanisms by which ANO1 is regulated by CFTR is yet to be investigated, but the small influx of chloride through CFTR seems to have some function since the ANO1 current was inhibited by the CFTR-specific open-channel blocker Gly-H 101 (Figure 3F). The fact that ANO1 is calcium activated [41] is consistent with a role of this channel in exocytosis. It can be argued that the increase in cAMP will enhance the voltage-dependent calcium-influx [58] and thereby increase the ANO1 current and insulin secretion. This process can most likely occur in parallel with the cAMP-dependent regulation of ANO1 via CFTR as demonstrated here. Our data, however, demonstrate a role for CFTR in regulating insulin secretion through a direct effect on exocytosis.

## Conclusions

**Figure 6:**
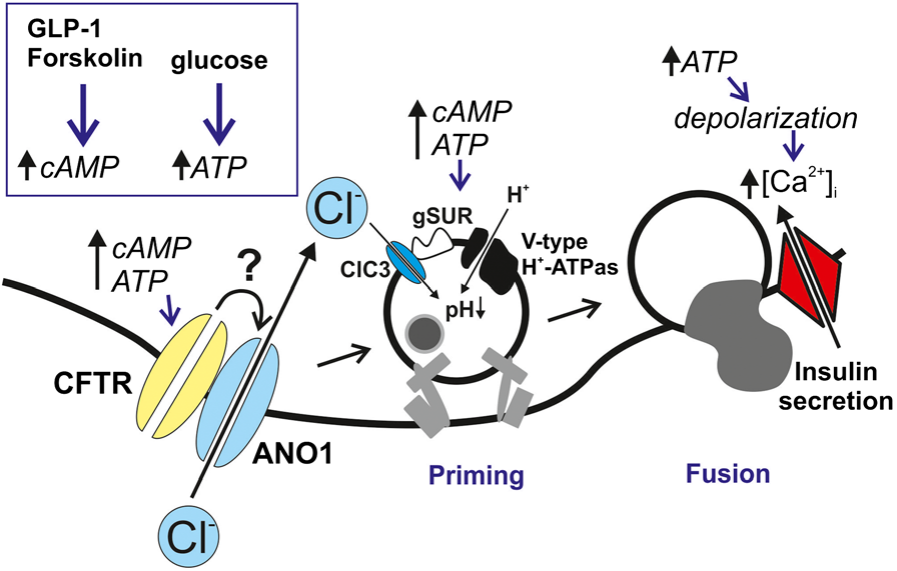
**Model describing a possible involvement of CFTR in beta-cell granular priming and exocytosis.** High glucose increases intracellular ATP leading to depolarization and influx of intracellular calcium triggering exocytosis. GLP-1 and forskolin (FSK) increase intracellular cAMP, which, together with the increase in ATP, enhance the priming of insulin granules, a process that involves influx of chloride into the granules through the intragranular ClC3 channels. Our data indicate that cAMP and ATP in parallel activate CFTR. We suggest that CFTR through a still unknown mechanism regulates ANO1 and hypothesize that the influx of chloride through ANO1 supplies the intragranular ClC3 channel and thereby enhances granular priming. CFTR, Cystic Fibrosis Transmembrane conductance regulator; ANO1, Anoctamin 1 chloride channel; gSUR, granular sulfonylurea receptor; Ca^2+^, calcium.

As illustrated in the model in Figure 6, we propose participation of CFTR, ANO1 and ClC3 in the same process and suggest that ion-fluxes through these ion channels together contribute to improved beta-cell exocytosis and priming. We hypothesize that cAMP activates CFTR, in addition to parallel activation of proteins already known to be important for insulin granule exocytosis. These include PKA that is stimulating calcium influx [58] and granular mobilization [49] and Epac2, which are involved in granular priming [19]. Epac2 is in complex with SUR1 and the granular ClC3 chloride channel [19,54]. We postulate that CFTR via ANO1 provides granular ClC3 chloride channels with chloride ions necessary to improve cAMP-dependent granular priming, exocytosis and secretion. This is possible since the flux of chloride ions changes during an action potential and is inward above the equilibrium potential for chloride. Thus, the direction of the chloride flux is into the cell at potentials needed to initiate exocytosis (above approximately −20 mV).

Depolarization-evoked exocytosis of primed granules is hypothesized to correspond to the first phase insulin secretion [46], suggesting an important role for CFTR at this stage. Indeed, many CF patients suffer from postprandial hyperglycemia although they have normal fasting plasma glucose levels indicating that the beta-cells fail to respond upon increased insulin demand. For these patients, impaired insulin secretion is mostly apparent during the first phase, strongly indicating a defect at the level of the pancreatic beta-cell [9,14-16]. The impaired insulin secretion has been suggested to be due to severe exocrine tissue damage destroying the beta-cells [4,10,11,13,15], but others have indicated direct effects on beta-cell function [9,15,16,60,61]. Indeed, reduced or absent acute insulin response to glucose in patients with CFRD was recently shown to be compensated by pharmacological CFTR potentiation [16]. Here we provide a likely explanation to the clinical observations supporting an effect on beta-cell function. We demonstrate for the first time that CFTR has a role at the cellular level, in both human and mouse beta-cells, by regulating insulin secretion. Specifically, we provide evidence that interference with CFTR affects cAMP-dependent rapid exocytosis of primed granules important for first phase insulin release.

## Abbreviations

ABC transporters: ATP-binding cassette-transporters; ANO1: Anoctamin 1 chloride channel; BMI: Body Mass Index; CF: Cystic fibrosis; CFRD: Cystic fibrosis-related diabetes; CFTR: Cystic fibrosis transmembrane conductance regulator; Epac2: Exchange protein directly activated by cAMP 2; FSK: Forskolin; gSUR: granular Sulfonylura receptor; MSD: Membrane spanning domain; NBD: Nucleotide binding domain; NS: surface density of granules; Nv: volume density of granules; RIA: Radioimmunoassay; SNARE: Soluble NSF attachment protein receptor; SUR: Sulfonylurea receptor; TEM: Transmission electron microscopy; VRAC: Volume regulated anion channel.

## Competing interests

The authors declare they have no competing interests.

## Authors’ contribution

AE and LE designed the project, performed research, analyzed the data and wrote the paper. JLSE performed research, analyzed data and reviewed and revised the manuscript. AW analyzed data and reviewed and revised the manuscript. MF-T designed the project and wrote the paper. All authors read and approved the final manuscript.

## References

[B1] Sheppard DN Welsh MJ Structure and function of the CFTR chloride channel Physiol Rev 1999 79 S23 S45 9922375 10.1152/physrev.1999.79.1.S23

[B2] Schwiebert EM Benos DJ Egan ME Stutts MJ Guggino WB CFTR is a conductance regulator as well as a chloride channel Physiol Rev 1999 79 S145 S166 9922379 10.1152/physrev.1999.79.1.S145

[B3] Rowntree RK Harris A The phenotypic consequences of CFTR mutations Ann Hum Genet 2003 67 471 485 10.1046/j.1469-1809.2003.00028.x 12940920

[B4] Brennan AL Geddes DM Gyi KM Baker EH Clinical importance of cystic fibrosis-related diabetes J Cyst Fibros 2004 3 209 222 10.1016/j.jcf.2004.08.001 15698938

[B5] de Valk H van der Graaf E Cystic Fibrosis-Related Diabetes in adults: where can we go from here? Rev Diabet Stud 2007 4 6 12 10.1900/RDS.2007.4.6 17565411 PMC1892524

[B6] Dobson L Sheldon CD Hattersley AT Understanding cystic-fibrosis-related diabetes: best thought of as insulin deficiency? J R Soc Med 2004 97 26 35 10.1258/jrsm.97.1.26 15239291 PMC1308796

[B7] Moran A Dunitz J Nathan B Saeed A Holme B Thomas W Cystic fibrosis-related diabetes: current trends in prevalence, incidence, and mortality Diabetes Care 2009 32 1626 1631 10.2337/dc09-0586 19542209 PMC2732133

[B8] Moran A Diem P Klein DJ Levitt MD Robertson RP Pancreatic endocrine function in cystic fibrosis J Pediatr 1991 118 715 723 10.1016/S0022-3476(05)80032-0 2019925

[B9] Kelly A Moran A Update on cystic fibrosis-related diabetes J Cyst Fibros 2013 12 318 331 10.1016/j.jcf.2013.02.008 23562217

[B10] Abdul-Karim FW Dahms BB Velasco ME Rodman HM Islets of Langerhans in adolescents and adults with cystic fibrosis. A quantitative study Arch Pathol Lab Med 1986 110 602 606 2872872

[B11] Iannucci A Mukai K Johnson D Burke B Endocrine pancreas in cystic fibrosis: an immunohistochemical study Hum Pathol 1984 15 278 284 10.1016/S0046-8177(84)80191-4 6365738

[B12] Löhr M Goertchen P Nizze H Gould NS Gould VE Oberholzer M Heitz PU Klöppel G Cystic fibrosis associated islet changes may provide a basis for diabetes. An immunocytochemical and morphometrical study Virchows Arch A Pathol Anat Histopathol 1989 414 179 185 10.1007/BF00718598 2492695

[B13] Soejima K Landing BH Pancreatic islets in older patients with cystic fibrosis with and without diabetes mellitus: morphometric and immunocytologic studies Pediatr Pathol 1986 6 25 46 10.3109/15513818609025923 2881283

[B14] Lanng S Glucose intolerance in cystic fibrosis patients Paediatr Respir Rev 2001 2 253 259 10.1053/prrv.2001.0148 12052327

[B15] Moran A Becker D Casella SJ Gottlieb PA Kirkman MS Marshall BC Slovis B Epidemiology, pathophysiology, and prognostic implications of cystic fibrosis-related diabetes: a technical review Diabetes Care 2010 33 2677 2683 10.2337/dc10-1279 21115770 PMC2992212

[B16] Bellin MD Laguna T Leschyshyn J Regelmann W Dunitz J Billings J Moran A Insulin secretion improves in cystic fibrosis following ivacaftor correction of CFTR: a small pilot study Pediatr Diabetes 2013 14 417 421 10.1111/pedi.12026 23952705 PMC3804832

[B17] Kinard TA Satin LS An ATP-sensitive Cl- channel current that is activated by cell swelling, cAMP, and glyburide in insulin-secreting cells Diabetes 1995 44 1461 1466 10.2337/diab.44.12.1461 7589855

[B18] Boom A Lybaert P Pollet JF Jacobs P Jijakli H Golstein PE Sener A Malaisse WJ Beauwens R Expression and localization of cystic fibrosis transmembrane conductance regulator in the rat endocrine pancreas Endocrine 2007 32 197 205 10.1007/s12020-007-9026-x 18040894

[B19] Eliasson L Ma X Renström E Barg S Berggren PO Galvanovskis J Gromada J Jing X Lundquist I Salehi A Sewing S Rorsman P SUR1 regulates PKA-independent cAMP-induced granule priming in mouse pancreatic B-cells J Gen Physiol 2003 121 181 197 10.1085/jgp.20028707 12601083 PMC2217330

[B20] Esguerra JL Bolmeson C Cilio CM Eliasson L Differential glucose-regulation of microRNAs in pancreatic islets of non-obese type 2 diabetes model Goto-Kakizaki rat PLoS One 2011 6 e18613 10.1371/journal.pone.0018613 21490936 PMC3072418

[B21] Vikman J Ma X Hockerman GH Rorsman P Eliasson L Antibody inhibition of synaptosomal protein of 25 kDa (SNAP-25) and syntaxin 1 reduces rapid exocytosis in insulin-secreting cells J Mol Endocrinol 2006 36 503 515 10.1677/jme.1.01978 16720719

[B22] De Marinis YZ Salehi A Ward CE Zhang Q Abdulkader F Bengtsson M Braha O Braun M Ramracheya R Amisten S Habib AM Moritoh Y Zhang E Reimann F Rosengren AH Shibasaki T Gribble F Renström E Seino S Eliasson L Rorsman P GLP-1 inhibits and adrenaline stimulates glucagon release by differential modulation of N- and L-type Ca2+ channel-dependent exocytosis Cell Metab 2010 11 543 553 10.1016/j.cmet.2010.04.007 20519125 PMC4310935

[B23] Caci E Caputo A Hinzpeter A Arous N Fanen P Sonawane N Verkman AS Ravazzolo R Zegarra-Moran O Galietta LJ Evidence for direct CFTR inhibition by CFTR(inh)-172 based on Arg347 mutagenesis Biochem J 2008 413 135 142 10.1042/BJ20080029 18366345

[B24] Muanprasat C Sonawane ND Salinas D Taddei A Galietta LJ Verkman AS Discovery of glycine hydrazide pore-occluding CFTR inhibitors: mechanism, structure-activity analysis, and *in vivo* efficacy J Gen Physiol 2004 124 125 137 10.1085/jgp.200409059 15277574 PMC2229623

[B25] Akesson B Henningsson R Salehi A Lundquist I Islet constitutive nitric oxide synthase and glucose regulation of insulin release in mice J Endocrinol 1999 163 39 48 10.1677/joe.0.1630039 10495405

[B26] Andersson SA Pedersen MG Vikman J Eliasson L Glucose-dependent docking and SNARE protein-mediated exocytosis in mouse pancreatic alpha-cell Pflugers Arch 2011 462 443 454 10.1007/s00424-011-0979-5 21643653

[B27] Tian G Sandler S Gylfe E Tengholm A Glucose- and hormone-induced cAMP oscillations in alpha- and beta-cells within intact pancreatic islets Diabetes 2011 60 1535 1543 10.2337/db10-1087 21444924 PMC3292328

[B28] Tian G Sol ER Xu A Tengholm A Prolonged exposure to palmitate deteriorates glucose-induced cAMP generation and pulsatile insulin secretion Diabetologia 2013 S56 S194

[B29] Cabrera O Berman DM Kenyon NS Ricordi C Berggren PO Caicedo A The unique cytoarchitecture of human pancreatic islets has implications for islet cell function Proc Natl Acad Sci U S A 2006 103 2334 2339 10.1073/pnas.0510790103 16461897 PMC1413730

[B30] Huypens P Ling Z Pipeleers D Schuit F Glucagon receptors on human islet cells contribute to glucose competence of insulin release Diabetologia 2000 43 1012 1019 10.1007/s001250051484 10990079

[B31] Seino S Cell signalling in insulin secretion: the molecular targets of ATP, cAMP and sulfonylurea Diabetologia 2012 55 2096 2108 10.1007/s00125-012-2562-9 22555472

[B32] Gopel SO Kanno T Barg S Weng XG Gromada J Rorsman P Regulation of glucagon release in mouse -cells by KATP channels and inactivation of TTX-sensitive Na + channels J Physiol 2000 528 509 520 10.1111/j.1469-7793.2000.00509.x 11060128 PMC2270147

[B33] Gabriel SE Clarke LL Boucher RC Stutts MJ CFTR and outward rectifying chloride channels are distinct proteins with a regulatory relationship Nature 1993 363 263 268 10.1038/363263a0 7683773

[B34] Cliff WH Schoumacher RA Frizzell RA cAMP-activated Cl channels in CFTR-transfected cystic fibrosis pancreatic epithelial cells Am J Physiol 1992 262 C1154 C1160 1375432 10.1152/ajpcell.1992.262.5.C1154

[B35] Schultz BD Singh AK Devor DC Bridges RJ Pharmacology of CFTR chloride channel activity Physiol Rev 1999 79 S109 S144 9922378 10.1152/physrev.1999.79.1.S109

[B36] Gojkovic-Bukarica L Hambrock A Loffler-Walz C Quast U Russ U Mg2+ sensitizes KATP channels to inhibition by DIDS: dependence on the sulphonylurea receptor subunit Br J Pharmacol 2002 137 429 440 10.1038/sj.bjp.0704905 12359624 PMC1573525

[B37] Sheppard DN Welsh MJ Effect of ATP-sensitive K + channel regulators on cystic fibrosis transmembrane conductance regulator chloride currents J Gen Physiol 1992 100 573 591 10.1085/jgp.100.4.573 1281220 PMC2229110

[B38] Henquin JC Dufrane D Nenquin M Nutrient control of insulin secretion in isolated normal human islets Diabetes 2006 55 3470 3477 10.2337/db06-0868 17130494

[B39] Ishiyama N Ravier MA Henquin JC Dual mechanism of the potentiation by glucose of insulin secretion induced by arginine and tolbutamide in mouse islets Am J Physiol Endocrinol Metab 2006 290 E540 E549 16249257 10.1152/ajpendo.00032.2005

[B40] Kunzelmann K Tian Y Martins JR Faria D Kongsuphol P Ousingsawat J Wolf L Schreiber R Airway epithelial cells–functional links between CFTR and anoctamin dependent Cl- secretion Int J Biochem Cell Biol 2012 44 1897 1900 10.1016/j.biocel.2012.06.011 22710346

[B41] Winpenny JP Gray MA The anoctamin (TMEM16) gene family: calcium-activated chloride channels come of age Exp Physiol 2012 97 175 176 22302790 10.1113/expphysiol.2011.058214

[B42] Malaisse WJ Virreira M Zhang Y Crutzen R Bulur N Lybaert P Golstein PE Sener A Beauwens R Role of anoctamin 1 (TMEM16A) as a volume regulated anion channel in insulin-producing cells Diabetologia 2012 55 S204 10.1007/s00125-011-2328-9

[B43] Mahdi T Hänzelmann S Salehi A Muhammed SJ Reinbothe TM Tang Y Axelsson AS Zhou Y Jing X Almgren P Krus U Taneera J Blom AM Lyssenko V Esguerra JL Hansson O Eliasson L Derry J Zhang E Wollheim CB Groop L Renström E Rosengren AH Secreted frizzled-related protein 4 reduces insulin secretion and is overexpressed in type 2 diabetes Cell Metab 2012 16 625 633 10.1016/j.cmet.2012.10.009 23140642

[B44] Namkung W Phuan PW Verkman AS TMEM16A inhibitors reveal TMEM16A as a minor component of calcium-activated chloride channel conductance in airway and intestinal epithelial cells J Biol Chem 2011 286 2365 2374 10.1074/jbc.M110.175109 21084298 PMC3023530

[B45] Ashcroft FM Rorsman P Diabetes mellitus and the beta cell: the last ten years Cell 2012 148 1160 1171 10.1016/j.cell.2012.02.010 22424227 PMC5890906

[B46] Eliasson L Abdulkader F Braun M Galvanovskis J Hoppa MB Rorsman P Novel aspects of the molecular mechanisms controlling insulin secretion J Physiol 2008 586 3313 3324 10.1113/jphysiol.2008.155317 18511483 PMC2538808

[B47] Barg S Huang P Eliasson L Nelson DJ Obermuller S Rorsman P Thevenod F Renstrom E Priming of insulin granules for exocytosis by granular Cl(−) uptake and acidification J Cell Sci 2001 114 2145 2154 11493650 10.1242/jcs.114.11.2145

[B48] Eliasson L Renstrom E Ding WG Proks P Rorsman P Rapid ATP-dependent priming of secretory granules precedes Ca(2+)-induced exocytosis in mouse pancreatic B-cells J Physiol 1997 503 399 412 10.1111/j.1469-7793.1997.399bh.x 9306281 PMC1159871

[B49] Renstrom E Eliasson L Rorsman P Protein kinase A-dependent and -independent stimulation of exocytosis by cAMP in mouse pancreatic B-cells J Physiol 1997 502 105 118 10.1111/j.1469-7793.1997.105bl.x 9234200 PMC1159575

[B50] Ganeshan R Di A Nelson DJ Quick MW Kirk KL The interaction between syntaxin 1A and cystic fibrosis transmembrane conductance regulator Cl- channels is mechanistically distinct from syntaxin 1A-SNARE interactions J Biol Chem 2003 278 2876 2885 10.1074/jbc.M211790200 12446681

[B51] Naren AP Nelson DJ Xie W Jovov B Pevsner J Bennett MK Benos DJ Quick MW Kirk KL Regulation of CFTR chloride channels by syntaxin and Munc18 isoforms Nature 1997 390 302 305 10.1038/36882 9384384

[B52] Best L Glucose-induced electrical activity in rat pancreatic beta-cells: dependence on intracellular chloride concentration J Physiol 2005 568 137 144 10.1113/jphysiol.2005.093740 16024506 PMC1474780

[B53] Braun M Ramracheya R Bengtsson M Clark A Walker JN Johnson PR Rorsman P Gamma-aminobutyric acid (GABA) is an autocrine excitatory transmitter in human pancreatic beta-cells Diabetes 2010 59 1694 1701 10.2337/db09-0797 20413510 PMC2889769

[B54] Li DQ Jing X Salehi A Collins SC Hoppa MB Rosengren AH Zhang E Lundquist I Olofsson CS Mörgelin M Eliasson L Rorsman P Renström E Suppression of sulfonylurea- and glucose-induced insulin secretion in vitro and in vivo in mice lacking the chloride transport protein ClC-3 Cell Metab 2009 10 309 315 10.1016/j.cmet.2009.08.011 19808023

[B55] Best L Brown PD Sener A Malaisse WJ Electrical activity in pancreatic islet cells: the VRAC hypothesis Islets 2010 2 59 64 10.4161/isl.2.2.11171 21099297

[B56] Henquin JC Meissner HP Dibutyryl cyclic AMP triggers Ca2+ influx and Ca2 + −dependent electrical activity in pancreatic B cells Biochem Biophys Res Commun 1983 112 614 620 10.1016/0006-291X(83)91508-5 6303325

[B57] Ikeuchi M Cook DL Glucagon and forskolin have dual effects upon islet cell electrical activity Life Sci 1984 35 685 691 10.1016/0024-3205(84)90264-9 6087071

[B58] Ammala C Ashcroft FM Rorsman P Calcium-independent potentiation of insulin release by cyclic AMP in single beta-cells Nature 1993 363 356 358 10.1038/363356a0 7684514

[B59] Ammala C Eliasson L Bokvist K Larsson O Ashcroft FM Rorsman P Exocytosis elicited by action potentials and voltage-clamp calcium currents in individual mouse pancreatic B-cells J Physiol 1993 472 665 688 8145165 10.1113/jphysiol.1993.sp019966 PMC1160506

[B60] Battezzati A Mari A Zazzeron L Alicandro G Claut L Battezzati PM Colombo C Identification of insulin secretory defects and insulin resistance during oral glucose tolerance test in a cohort of cystic fibrosis patients Eur J Endocrinol 2011 165 69 76 10.1530/EJE-10-1003 21502328

[B61] Olivier AK Yi Y Sun X Sui H Liang B Hu S Xie W Fisher JT Keiser NW Lei D Zhou W Yan Z Li G Evans TI Meyerholz DK Wang K Stewart ZA Norris AW Engelhardt JF Abnormal endocrine pancreas function at birth in cystic fibrosis ferrets J Clin Invest 2012 122 3755 3768 10.1172/JCI60610 22996690 PMC3534166

